# Lowering the apoptotic threshold in colorectal cancer cells by targeting mitochondria

**DOI:** 10.1186/1475-2867-10-31

**Published:** 2010-09-06

**Authors:** Jayesh Sagar, Kevin Sales, Jan-Willem Taanman, Sas Dijk, Marc Winslet

**Affiliations:** 1 Division of Surgery and Interventional Science, University College London, Gower Street, London, WC1E 6BT, UK; 2 Academic Department of Surgery, Royal Free & University College Medical School, Pond Street, London, NW3 2QG, UK; 3 Department of Clinical Neuroscience, Royal Free & University College Medical School, Pond Street, London, NW3 2QG, UK

## Abstract

**Background:**

Colorectal cancer is the third most-common cancer and the second most-common cause of cancer related death in UK. Although chemotherapy plays significant role in the treatment of colorectal cancer, morbidity and mortality due to drug resistance and cancer metastasis are yet to be eliminated. Recently, doxycycline has been reported to have cytotoxic and anti-proliferating properties in various cancer cells. In this study, whether doxycycline was apoptosis threshold lowering agent in colorectal cancer cells by targeting mitochondria was answered.

**Results:**

This study showed dose-dependent cytotoxic effects of cisplatin, oxaliplatin and doxycycline in HT29 colorectal cancer cells. Doxycycline showed inhibition of cytochrome-*c-*oxidase activity in these cells over a time-period. The pre-treatment of doxycycline reported statistically significant increased cytotoxicity of cisplatin and oxaliplatin compared to cisplatin and oxaliplatin alone. The caspase studies revealed significantly less expression and activity of caspase 3 in HT29 cells pre-treated with doxycycline compared to the cells treated with cisplatin and oxaliplatin alone.

**Conclusions:**

It was concluded that doxycycline lowered the apoptotic threshold in HT 29 cells by targeting mitochondria. This also raised possible caspase-independent mechanisms of apoptosis in HT29 cells when pre-treated with doxycycline however this needs further research work.

## Background

Tetracyclines (TCNs) have long been used widely in clinical practice as antibiotics in various bacterial, mycoplasma, chlamydiae, rickettsiae and protozoan infections. Their main mechanism of action involves inhibition of protein synthesis by restricting binding of aminoacyl t-RNA to 30 S ribosomes. TCNs are believed to interfere in mitochondrial protein synthesis that let to the discovery of other effects of TCNs independent of their antimicrobial actions[1]. Recently, a renewed interest in study of TCNs has evolved due to their ability to inhibit matrix metalloproteinases (MMPs) in various cancers such as prostate[2], melanoma[3], osteosarcoma[4], breast[5], leukaemia [6] and colorectal cancers[7]. Some of TCNs have been shown to work as apoptotic inducers[8]. Despite TCNs' emerging role as anti-invasive and anti-proliferative drugs in cancer treatment, their apoptotic mechanisms are yet to be precisely defined.

Apoptosis is the mechanism by which chemotherapeutic agents induce cancer cell death[9]. There are two main mechanisms of apotptosis; the intrinsic and extrinsic pathways. Caspases, the proteolyic enzymes, cysteine proteases, play an essential role in execution of apoptosis[10]. In the extrinsic pathway, upon apoptotic stimulus, the death receptors bind to adaptor proteins and pro-caspases forming death initiating signal complex (DISC), which either lead to activation of caspases or cleavage of Bcl-2 family members causing apoptosis and cell death[11,12]. In the intrinsic pathway, the apoptotic stimulus activates Bcl-2 family members' protein synthesis, which causes cytochrome c release from mitochondria. The released cytochrome c, along with adaptor protein and pro-caspases forms an apoptosome and leads to release of activated caspases inducing apoptosis and cell death[11,12].

Colorectal cancer is the third most common cancer in males and the second most common cancer in females. It accounts for 9.7% of all the new cancers diagnosed and contributes 8.4% of cancer mortality worldwide[13]. Apart from the surgical option, chemotherapy and radiotherapy also play significant roles in the treatment of the colorectal cancer especially in advanced or metastatic disease. Different chemical agents including platinum agents have been used in various trials to improve the outcome of colorectal cancer treatment however problems of metastasis and recurrence are yet to be eliminated.

Doxycycline, one of the most widely used TCNs in clinical practice, is a long acting orally administered chemical agent. Apart from its antibiotic properties, it has been shown to inhibit cell proliferation and invasion and to induce apoptosis in colorectal cancer cells[14,15]. As doxycycline restricts oxidative phosphorylation by inhibition of mitochondrial protein synthesis and thus reduces ATP synthesis, the effects of combination therapy of doxycycline with cisplatin and oxaliplatin on HT 29 colorectal cancer cell line were investigated in this study in an attempt to improve the drug efficacy and to reduce drug resistance.

## Results

### Cytotoxicity of Drugs

**Figure 1:**
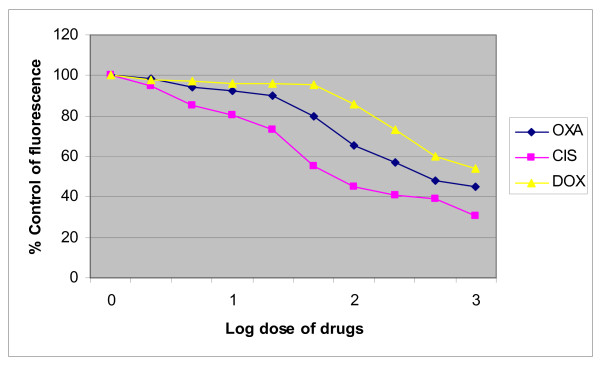
**Dose response relationship of cisplatin, oxaliplatin and doxycycline**. 2 × 10^4 ^HT 29 cells/ml were treated with various concentrations (1 to 1000 micromolar) of cisplatin, oxaliplatin and doxycycline (0.1 to 100 micrograms/ml) for 24 hours in the wells of 24 well plates and the cytotoxicity measured with Alamar blue assay according to the manufacturer's instructions. Control was taken as untreated cells. Data are depicted as means of six experiments ± standard deviation.

The cytotoxicity studies revealed dose dependent cytotoxic effects of cisplatin, oxaliplatin and doxycycline on HT 29 cells; however the effect of doxycycline was much lower compared to the platinum compounds (figure 1).

### Combination Treatment

**Figure 2:**
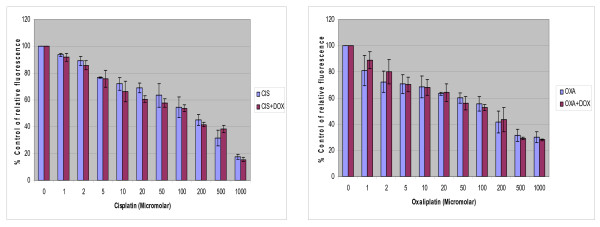
**Dose response relationship of combination treatment of doxycycline with platinum compounds**. 2 × 10^4 ^HT 29 cells/ml were treated in the wells of 24 well plates with cisplatin or oxaliplatin with and without 10 μg/ml of doxycycline for 24 hours. The cytotoxicity was assessed with Alamar blue assay according to the manufacturer's instructions. Data are depicted as means of six experiments ± standard deviation. (*p *= 0.84 for cisplatin and *p *= 0.95 for oxaliplatin experiments).

The combination treatment of doxycycline with platinum compounds in HT29 cells did not reveal any statistically significant difference in the cytotoxicity compared to the treatment of platinum compounds alone following 24 hours of treatment (Figure 2).

### Cytochrome c oxidase Staining

**Figure 3:**
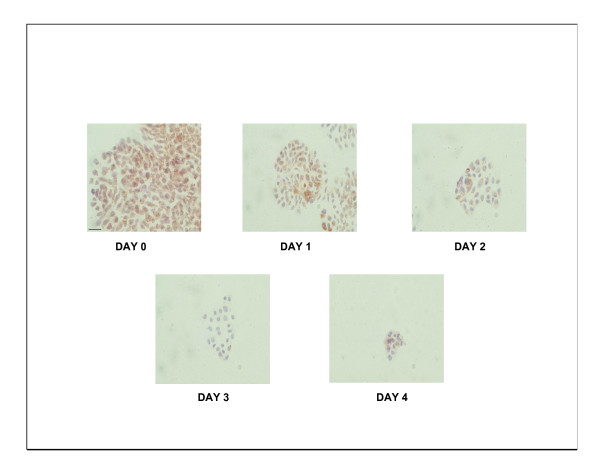
**Cytochrome *c *oxidase activity**. 2 × 10^4 ^HT 29 cells were treated with 10 μg/ml of doxycycline over a period of 4 days and staining for cytochrome *c *oxidase activity was performed as mentioned in methods. The brown staining is indicative of cytochrome *c *oxidase activity, while purple staining is indicative of cell nucleus. Representative micrographs of six independent experiments are shown. Scale bar equals 20 μM.

**Figure 4:**
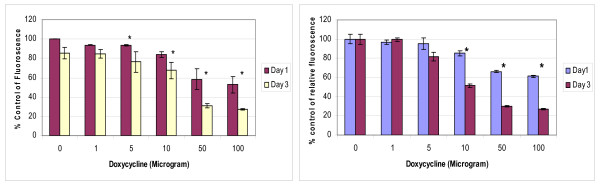
**Comparative cytotoxicity and anti-proliferative effects of doxycycline**. 2 × 10^4 ^HT 29 cells/ml were treated with doxycycline for 24 hours and 3 days in the wells of 6 well plates. The cytotoxicity and cell proliferation studies were performed with Alamar blue assay and Picogreen assay according to the manufacturer's instructions respectively. Data are depicted as means of six experiments ± standard deviation. (* - Denotes statistically significant difference in the activity).

Cytochrome *c *oxidase activity staining showed a significant lack of enzyme activity from day 3 onwards (Figure 3) in HT29 cells treated with doxycycline. Following the results of this experiment, cytotoxicity and cell proliferation studies were performed after 3 days of doxycycline treatment and they revealed statistically significant increased cytotoxicity and anti-prolifetative effects in HT29 cells after 3 days of doxycycline treatment compared to 24 hours of treatment (Figure 4).

### Pre-treatment of Doxycycline

**Figure 5:**
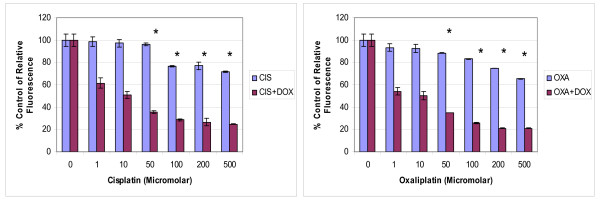
**Pre-treatment of doxycycline**. 2 × 10^4 ^HT 29 cells/ml were treated with 10 μg/ml of doxycycline for 3 days in the wells of 6 well plates followed by treatment with different concentrations of cisplatin and oxaliplatin for 24 hours. In similar settings, another set of experiments was established without pre-treatment of doxycycline for 3 days. The cytotoxicity was assessed with Alamar blue assay according to the manufacturer's instructions. Data are depicted as means of six experiments ± standard deviation. (*p *= 0.003 for cisplatin and *p *= 0.003 for oxaliplatin experiments). (* - Denotes statistically significant difference in the activity).

The cytotoxicity studies reported that 3 days pre-treatment of doxycycline enhanced the cytotoxic effects of cisplatin and oxaliplatin in HT29 cancer cells significantly compared to the treatment of platinum compounds alone (figure 5). This difference in the cytotoxicity was statistically significant.

### Caspase 3 Expression and Activity

**Figure 6:**
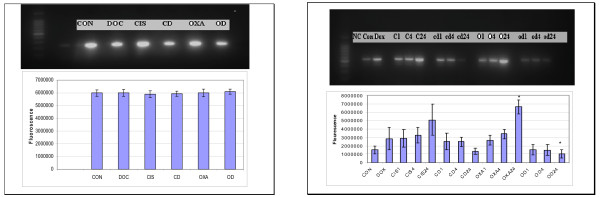
**Caspase 3 gene expression**. Gel electrophoresis of GAPDH amplicons is displayed as control on the left side of figure. (CON = Control, DOC = Doxycycline, CIS = Cisplatin, CD = Cisplatin and Doxycycline, OXA = Oxaliplatin, OD = Oxaliplatin and Doxycycline). The right side of figure displays gel electrophoresis of caspase 3. (NC = Negative control, Con = CON = Positive control, Dox = DOX = Doxycycline, C(CIS)1; C4; C24 = Cisplatin treatment for 1, 4 and 24 hours respectively, cd(CD)1; cd4; cd24 = Cisplatin and Doxycycline treatment for 1, 4 and 24 hours respectively, O(OXA)1; O4; O24 = Oxaliplatin treatment for 1, 4 and 24 hours respectively, od(OD)1; od4; 0d24 = Oxaliplatin and Doxycycline treatment for 1, 4 and 24 hours respectively). Data are depicted as means of six experiments ± standard deviation. (* - Denotes statistically significant difference in the activity).

**Figure 7:**
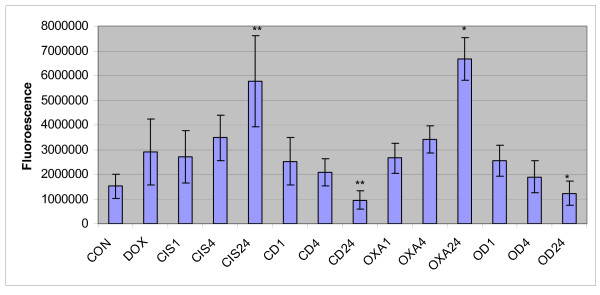
**Real time PCR for caspase 3 gene expression**. This figure displays quantitative presentation of caspase 3 gene expression in HT 29 cells by real time PCR. (CON = control, DOX = doxycycline, CIS1; 4; 24 = Cisplatin treatment for 1, 4 and 24 hours respectively, CD1; 4; 24 = Cisplatin and Doxycycline treatment for 1, 4 and 24 hours respectively, OXA1; 4; 24 = Oxaliplatin treatment for 1, 4 and 24 hours respectively, OD1; 4; 24 = Oxaliplatin and Doxycycline treatment for 1, 4 and 24 hours respectively). Data are depicted as means of six experiments ± standard deviation. (* - Denotes statistically significant difference in the activity).

**Figure 8:**
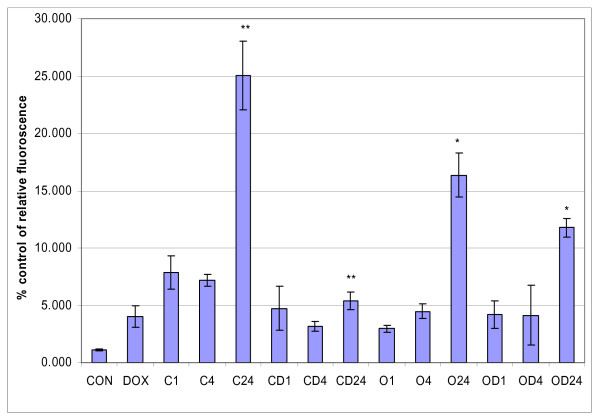
**Caspase 3 activity**. This figure displays quantitative assessment of caspase 3 activity in HT 29 cells following drug treatments. (CON = control, DOX = doxycycline, C1; 4; 24 = Cisplatin treatment for 1, 4 and 24 hours respectively, CD1; 4; 24 = Cisplatin and Doxycycline treatment for 1, 4 and 24 hours respectively, O1; 4; 24 = Oxaliplatin treatment for 1, 4 and 24 hours respectively, OD1; 4; 24 = Oxaliplatin and Doxycycline treatment for 1, 4 and 24 hours respectively). Data are depicted as means of six experiments ± standard deviation. (* - Denotes statistically significant difference in the activity).

Caspase 3 gene expression studies revealed increased caspase 3 gene expression in a time dependent manner in HT29 cells treated with cisplatin and oxaliplatin, but caspase 3 transcript levels decreased in a time dependent manner in the cells pre-treated with doxycycline (figure 6). As these experiments were performed with one-step RT-PCR (reverse transcription - polymerase chain reation) method, to verify these findings, the real time polymerase chain reactions (PCR) were used for the assessment of caspase 3 gene expression. Real time PCR showed similar findings to the results of RT PCR (figure 7). Caspase 3 activity study showed time dependent increase in caspase 3 activity in HT29 cells treated with platinum compounds but there were no such trend in HT29 cells pre-treated with doxycycline (figure 8).

## Discussion

Colorectal cancer constitutes the third most common cancer in UK. It is the second leading cause of cancer deaths in UK. About one in 20 develops colorectal cancer in their lifetime. About 16,000 people are dying from the colorectal cancer every year in UK (Cancer Statistics, Cancer Research UK 2005). Three main therapeutic options available for colorectal cancer; Surgery, Chemotherapy and Radiotherapy, are used either on their own or in combinations. The main reasons for mortality from colorectal cancer are metastases or recurrence. Recently, doxycycline has been shown to have MMPs inhibitory actions and thus has potential in prevention of metastases[14]. Apart from being an antibiotic, doxycycline has been reported to have cytotoxic actions and to suppress cell proliferation[14]. To date, various studies have reported the apoptotic effects of doxycycline in different cancers including prostate cancer[16], leukaemia[6], osteosarcoma[17] and pancreas cancer[18]. However, precise mechanisms of apoptosis are not well understood. Similarly, none of the work has investigated the impact of doxycycline with other platinum agents in colorectal cancer. This study answered whether doxycycline has any synergistic effects with platinum compounds; cisplatin and oxaliplatin, exploring its potential use in colorectal cancer therapy.

Results of this study showed cisplatin, oxaliplatin and doxycycline to be cytotoxic to HT 29 colorectal cancer cells following 24 hours of treatment in a dose-dependent manner; however, cisplatin and oxaliplatin were more cytotoxic than doxycycline (figure 1). To rule out any impact of serum in media, the cytotoxicity studies were performed using media with and without serum. It was found that the drugs were more cytotoxic in media containing serum, but this did not reach statistical significance (data not shown) and therefore future experiments were performed in the serum supplemented media. The combination treatment of doxycycline and platinum compounds in HT29 cells did not show any statistically significant difference in the cytotoxicity compared to cisplatin and oxaliplatin alone following 24 hours of treatments (figure 2). Thus in these initial experiments, our study failed to show any synergistic actions of doxycycline with platinum compounds. As these combinations did not show any difference in the cytotoxicity, the duration of treatment with doxycycline necessary to decrease the threshold for the cytotoxic effects of the cisplatin and oxaliplatin were looked in. As cytochrome *c *oxidase (complex IV) plays significant role in the oxidative phosphorylation of mitochondria and doxycycline works by inhibition of mitochondrial protein synthesis and thus inhibition of oxidative phosphorylation, cytochrome *c *oxidase activity was assessed over a period of time following doxycycline treatment. The cytochrome c oxidase activity reduced significantly from day 3 onwards in HT 29 cells following treatment of doxycycline (Figure 3). Following this finding, three days treatment of doxycycline of HT 29 cells showed significantly increased cytotoxic and anti-proliferative effects compared to 24 hours of treatment (figure 4). Thus it was decided to pre-treat HT 29 cells for 3 days before the treatment of platinum compounds to assess whether this would help to lower the apoptotic threshold. The cytotoxicity studies following 3 day pre-treatment of doxycycline revealed statistically significant higher cytotoxicity compared to cisplatin or oxaliplatin treatment alone (Figure 5). This suggested that doxycycline lowered the apoptotic threshold in colorectal cancer cells for the cytotoxic actions of cisplatin and oxaliplatin. Although doxycycline had shown apoptotic lowering potential, the precise mechanism of this effect was unclear. In an attempt to understand it, the caspase studies were performed. Caspases are the cysteine proteases and are crucial for the process of apoptosis. Caspase 3 is one of the executioner caspases involved towards the end of apoptotic induction following intrinsic as well as extrinsic pathways of apotptosis. Caspase 3 gene expression study revealed increased gene expression in a dose dependent manner in HT 29 cells treated with cisplatin and oxaliplatin but there was no increase in caspase 3 gene expression in the cells pre-treated with doxycycline (figure 6). These findings were confirmed by another method for assessment of caspase 3 gene expression (real time PCR studies) (figure 7). In order to verify these findings, it was decided to look at caspase 3 activity in these cells. The caspase 3 activity study reported similar findings to the caspase 3 gene expression study (figure 8). The reduction in the caspase 3 gene expression and activity in HT 29 cells pre-treated with doxycycline might be due to the time change in the measurement of gene expression and activity. However, caspase-independent mechanisms of apoptosis might be another explanation for these findings as few studies reported caspase-independent mechanisms of apoptosis in different cell lines[15]. Thus, this study suggested that pre-treatment of doxycycline lowered the apoptotic threshold in HT 29 colorectal cancer cells which might be due to caspase-independent mechanisms.

The advantage of doxycycline over other tetracyclines is its longer duration of action and comparatively less toxicity. Apart from cytotoxic and anti-proliferative properties of doxycycline, the key finding in this experiment was the inhibition of cytochrome c oxidase by doxycycline which suggested involvement of mitochondria in its actions. This study also suggests beneficial role of doxycycline pre-treatment in enhancing the cytotoxic effects of cisplatin and oxaliplatin with possible caspase independent mechanisms. As deficits or defects in the apoptotic pathways are frequently present in the tumours and are possible reasons for resistance to the chemotherapeutic agents, doxycycline may be useful in overcoming drug resistance however further research is required to evaluate the precise role of doxycycline in more detail before considering its potential use in clinical trails.

## Conclusions

We conclude that doxycycline may be useful as antiproliferative and cytotoxic agent and may be used to improve the efficacy of cisplatin and oxaliplatin in colorectal cancer cells. However, further studies are needed to clarify precise mechanisms of doxycycline in colorectal cancer cells.

## Methods

### Cell line

The human colorectal cancer cell line, HT 29 (ECACC, UK) was used in all experiments. The HT 29 cells were maintained in McCoy 5A media supplemented with 10% fetal bovine serum, penicillin (50 units/ml) and streptomycin (50 units/ml) at 37°C in a humidified atmosphere with 95% air and 5% CO_2 _.

### Chemical reagents

Cisplatin (Bristol-Myers Squibb, UK), oxaliplatin (Sanofi-Synthelabo, UK) and doxycycline (Alpharma, UK) were used in this study.

### Cell proliferation analysis

Cell proliferation was determined by Picogreen assay (Qiagen, UK) according to manufacturer's guidelines. 2 × 10^4 ^cells/ml HT 29 cells were plated in 6 well plates and treated with 1, 5, 10, 50 and 100 micrograms/ml (μg/ml) concentrations of doxycycline for 24 hours and 3 days respectively followed by cell proliferation studies.

### Cytotoxicity analysis

Cytotoxicity of drugs was determined by Alamar blue assay (Serotec, UK) according to manufacturer's guidelines. 2 × 10^4 ^cells/ml HT 29 cells were plated in 24 well plates and treated with different concentrations of cisplatin, oxaliplatin and doxycycline for 24 hours followed by cytotoxicity study. In similar settings, 2 × 10^4 ^cells/ml HT 29 cells were treated with combination of 10 μg/ml of doxcycline with different concentrations of cisplatin and oxaliplatin for 24 hours followed by cytotoxicity assay. In another experiment, 2 × 10^4 ^cells/ml HT 29 cells were plated in 6 well plates and treated with doxycycline for 3 days followed by cytotoxicity assay. In doxycycline pre-treatment experiment, 2 × 10^4 ^cells/ml HT 29 cells were plated in 6 well plates and then treated with different concentrations of cisplatin and oxaliplatin for 24 hours with or without 3 days pre-treatment of 10 μg/ml doxycycline followed by cytotoxicity assay.

### Cytochrome c oxidase activity

2 × 10^4 ^cells/ml HT 29 cells were plated on glass cover slips in 6 well plates for 24 hours and then treated with doxycycline over a period of 4 days. Cells were stained with 3,3' diaminobenzidine tetrahydrochloride (DAB) (Sigma, UK) staining according to guidelines and cytochrome *c *oxidase activity was visualised under a microscope.

### One Step RT-PCR and Real Time PCR for Caspase 3 gene expression

2 × 10^4 ^HT 29 cells/ml were cultured for 24 hours in the wells of 6 well plates followed by 3 day treatment of 10 μg/ml of doxycycline. At the end of 3 days, the cells were treated with different concentrations of cisplatin and oxaliplatin for 1, 4 and 24 hours. After respective treatment times, cells were trypsinized and cell pellets were collected by centrifuging at 500 rounds per minute (rpm) for 5 minutes. In a similar setting, the cells were cultured but without pre-treatment of doxycycline and at the end of day 3, cells were treated with cisplatin and oxaliplatin alone. At 1, 4 and 24 hours of treatment, the cell pellets were collected as mentioned earlier. The cell pellets were preserved at -80°C. These cell pellets were used for RNA extraction with the RNA-easy minikit (QIAGEN, UK). One step RT PCR was performed for caspase 3 gene expression using one-step RT PCR kit (QIAGEN, UK) and real time PCR was performed using two steps, first step of reverse transcription with the Omniscript RT Kit (Qiagen, UK) followed by real time PCR with Roche Molecular Lightcycler.

### Caspase 3 activity

2 × 10^4 ^HT 29 cells/ml were cultured for 24 hours in the wells of 6 well plates followed by 3 day treatment of 10 μg/ml of doxycycline. At the end of 3 days, the cells were treated with different concentrations of cisplatin and oxaliplatin for 1, 4 and 24 hours. After respective treatment times, caspase 3 activity was assessed using Caspase-3 Fluorometric Assay kit (R&D Systems, UK) according to manufacture's guidelines. In similar settings, HT 29 cells were treated with cisplatin and oxaliplatin without any pre-treatment of doxycycline followed by caspase 3 activity assays.

### Statistical Analysis

The t test with two samples was performed for statistical analysis. The obtained p values were used for interpretation of results. The p value of ≤ 0.05 was considered statistically significant. For the purpose of data analysis, the Microsoft XP system was used.

## Competing interests

The authors declare that they have no competing interests.

## Authors' contributions

JS - Responsible for the work as main research worker and preparation of this manuscript. KS - Responsible for cell culture work along with provision of the laboratory work. JWT - Responsible for immunohistochemical staining. SD - Responsible for caspase activity and PCR. MW - Responsible for overall supervision of this work and manuscript. All authors read and approved the final manuscript.
